# Monoprophylaxis With Cephalosporins for Transrectal Prostate Biopsy After the Fluoroquinolone-Era: A Multi-Institutional Comparison of Severe Infectious Complications

**DOI:** 10.3389/fonc.2021.684144

**Published:** 2021-06-10

**Authors:** Mike Wenzel, Jost von Hardenberg, Maria N. Welte, Samuel Doryumu, Benedikt Hoeh, Clarissa Wittler, Thomas Höfner, Maximilian C. Kriegmair, Maurice S. Michel, Felix KH. Chun, Jonas Herrmann, Philipp Mandel, Niklas Westhoff

**Affiliations:** ^1^ Department of Urology, University Hospital Frankfurt, Goethe University Frankfurt am Main, Frankfurt, Germany; ^2^ Cancer Prognostics and Health Outcomes Unit, Division of Urology, University of Montreal Health Center, Montreal, QC, Canada; ^3^ GeSRU Academics Prostate Cancer Working Group, Planegg, Germany; ^4^ Department of Urology and Urosurgery, University Medical Center Mannheim, Medical Faculty Mannheim, Heidelberg University, Mannheim, Germany; ^5^ Department of Urology, University Hospital Mainz, Mainz, Germany

**Keywords:** cephalosporins, urosepsis, urinary tract infections, biopsy, prostatic neoplasms, fluoroquinolones

## Abstract

**Background:**

To compare severe infectious complication rates after transrectal prostate biopsies between cephalosporins and fluoroquinolones for antibiotic monoprophylaxis.

**Material and Methods:**

In the multi-institutional cohort, between November 2014 and July 2020 patients received either cefotaxime (single dose intravenously), cefpodoxime (multiple doses orally) or fluoroquinolones (multiple-doses orally or single dose intravenously) for transrectal prostate biopsy prophylaxis. Data were prospectively acquired and retrospectively analyzed. Severe infectious complications were evaluated within 30 days after biopsy. Logistic regression models predicted biopsy-related infectious complications according to antibiotic prophylaxis, application type and patient- and procedure-related risk factors.

**Results:**

Of 793 patients, 132 (16.6%) received a single dose of intravenous cefotaxime and were compared to 119 (15%) who received multiple doses of oral cefpodoxime and 542 (68.3%) who received fluoroquinolones as monoprophylaxis. The overall incidence of severe infectious complications was 1.0% (n=8). No significant differences were observed between the three compared groups (0.8% *vs.* 0.8% *vs.* 1.1%, p=0.9). The overall rate of urosepsis was 0.3% and did not significantly differ between the three compared groups as well.

**Conclusion:**

Monoprophylaxis with third generation cephalosporins was efficient in preventing severe infectious complications after prostate biopsy. Single intravenous dose of cefotaxime and multiday regimen of oral cefpodoxime showed a low incidence of infectious complications <1%. No differences were observed in comparison to fluoroquinolones.

## Introduction

Prostate cancer, the most common cancer in men worldwide, is diagnosed with prostate biopsies (1). Currently, two different applicable biopsy approaches are available, a transrectal and transperineal approach (2). Several studies reported that cancer detection rates by systematic biopsies are comparable between both approaches (3, 4). However, regarding infectious rates, some studies suggest that the transperineal approach is associated with lower rates of infectious complications, while other report comparable infectious rates (4–6). Nonetheless, sufficient prospective data is lacking. In consequence, the transrectal approach is still used worldwide and guidelines do not recommend one approach over the other, since the advantage of the transrectal approach is the quick and easy performance in an outpatient setting under local anesthesia, while a transperineal biopsy is widely performed under general anesthesia (2, 4).

The U.S. Food and Drug Administration in 2018 and the European Medicine Association (EMA) in 2019 suspended the indication for fluoroquinolones as antibiotic prophylaxis due to its toxicity profile (7). In addition, as a consequence of increasing resistance rates, there were more severe infections during the past years after administration of fluoroquinolones (8). Since fluoroquinolones have been the antibiotic prophylaxis of choice for transrectal prostate biopsies for decades, no current guideline recommendation exists for other antimicrobial agents in prophylaxis (2, 9, 10). Cephalosporins represent an alternative as a monotherapeutic antibiotic prophylaxis. The application type, either as a single intravenous dose or multiple oral doses, has different advantages. However, although several studies investigated appropriate complication rates of other antibiotic regimes after transrectal prostate biopsy, these studies mostly focused on augmented regimes (11, 12). In consequence, studies comparing a monotherapeutic prophylaxis with cephalosporins *vs.* fluoroquinolones in a homogenous cohort are still pending (13).

We addressed this void and relied on a multi-institutional prostate biopsy database of two tertiary care university hospitals. We hypothesized that differences according to severe complication rates after transrectal prostate biopsy may not exist in the comparison of cephalosporins vs. fluoroquinolones. Moreover, we hypothesized that application form and duration of the antibiotic prophylaxis does not affect complication rates.

## Materials and Methods

### Study Population

The study was conducted in accordance with the Declaration of Helsinki. After ethic committee’s approval, patients who underwent a transrectal systematic prostate biopsy or combined magnetic resonance imaging (MRI)-targeted and transrectal systematic biopsy at either the Department of Urology, University Hospital Frankfurt (UKF), Germany or the Department of Urology and Urosurgery, University Medical Center Mannheim (UMM), Germany, between November 2014 and July 2020 were prospectively acquired and retrospectively analyzed. Exclusion criteria for the subsequent analyses were other antibiotic prophylaxis for transrectal prostate biopsies than cephalosporins or fluoroquinolones or augmented (combination of at least two antibiotic agents) antibiotic regimes (n=62). Indications for prostate biopsies were a primary cancer suspicion or patients under active surveillance in accordance with current guidelines (2).

### Transrectal Prostate Biopsy

According to the former institutional standards, at UMM, a urine culture was taken from every patient prior to prostate biopsies. During the study period, rectal swabs were not obtained by default. At UKF, urine cultures or rectal swabs were not performed on regular basis. Prior to transrectal prostate biopsy, a periprostatic local anesthesia was injected under ultrasound-guidance, as recommended (2). For systematic prostate biopsy, 12 cores (six cores from each prostate lobe) were taken according to current guidelines. For fusion biopsies, at least two cores were taken from each target, with high-end ultrasound machines (University Hospital Frankfurt: HiVison, Hitachi Medical Systems; University Medical Center Mannheim: Artemis™). In addition to targeted biopsy, systematic biopsy was performed in all patients.

### Antibiotic Prophylaxis and Follow-Up

All prostate biopsies were taken under antibiotic prophylaxis with a monotherapy with either cephalosporins or fluoroquinolones. The choice of an antibiotic regimen was based on the institutional standard at the time of biopsy. Since bioavailability may differ between the application types of both third generation cephalosporins, tabulation was made for cefotaxime (single dose intravenous application of 2g 20-60 minutes prior to biopsy) vs. cefpodoxime (multiple doses of 200mg oral application twice daily, beginning at least 24 hours prior to biopsy for five days according to current recommendations (14)). Due to the comparable bioavailability between oral and intravenous application, this stratification was not made for fluoroquinolones (intravenous or oral five-day application according to the historical fluoroquinolone standard). Patients at risk for an infectious endocarditis received an agent active against enterococci and were not included in this analysis. Patient and tumor characteristics, as well as severe infectious complication rates, defined as an emergency hospital consultation due to an UTI (according to current guidelines (14)) with or without fever, were collected from the patients’ hospital files within 30 days after prostate biopsy. Urosepsis was defined as previously described (15). During the pre-interventional briefing, patients were routinely instructed to inform a urologist in case of relevant complications.

### Statistical Analysis

Descriptive statistics included frequencies and proportions for categorical variables. Medians and interquartile ranges (IQR) were reported for continuously coded variables. The Chi-square test was used for statistical significance in proportions’ differences. The t-test and Kruskal-Wallis test examined the statistical significance of means’ and distributions’ differences.

All tests were two sided with a level of significance set at p<0.05 and R software environment for statistical computing and graphics (version 3.4.3) was used for all analyses.

## Results

### Patient and Procedure Characteristics

Overall, 793 patients were eligible for analyses. The median age was 66 years (IQR 61-72 years). Among all patients, 66.7% were biopsy naïve. A median number of 14 cores (IQR 13-15) per biopsy were obtained. In total, 36 patients (4.5%) had diabetes mellitus, 33 patients (4.2%) had at least three chronical diseases (defined as multimorbidity), 16 patients (2%) were immunosuppressed, nine patients (1.1%) had an indwelling catheter and six patients (0.8%) reported recurrent UTIs. In the group of patients who received multiple oral doses of cefpodoxime, significantly more patients had comorbidities (p<0.001). An UTI during the past twelve months was reported by nine patients (1.1%) and 50 patients received an antibiotic treatment for any cause within the last six months prior to biopsy (6.3%).

Significant differences between the subgroups of antibiotic regimens existed in the median number of cores per biopsy, a history of UTIs within the last 12 months and application of antibiotics within the last six months, as well as cancer detection rates (all p<0.05).

All patient characteristics and biopsy results are displayed in **Table 1**.

**Table 1:** Patient characteristics stratified by antibiotic prophylaxis for transrectal prostate biopsy.

Variable		Overall N=793	Cefotaxime single dose (intravenous) N=132 (16.6%)	Cefpodoxime multiple doses(oral) N=119 (15.0%)	Fluoroquinolones N=542 (68.3%)	P value
**Age, years**	Median (IQR)	66 (61-72)	66 (60-73)	66 (60-72)	67 (61-72)	0.7
**PSA, ng/ml**	Median (IQR)	7.3 (5.3-11.9)	7.0 (5.2-9.6)	7.4 (5.0-12.2)	7.4 (5.3-12.2)	0.1
**Prostate volume, ml**	Median (IQR)	50 (38-70)	58 (43-73)	50 (36-72)	50 (38-70)	0.8
**Biopsy positive, n (%)**	Yes	516 (65.1)	95 (72.0)	84 (70.6)	337 (62.2)	0.04
**Cores per biopsy**	Median (IQR)	14 (13-15)	16 (14-16)	13 (12-14)	14 (12-15)	<0.001
**Positive cores per biopsy**	Median (IQR)	2 (0-6)	3 (0-6)	3 (0-5)	2 (0-6)	0.2
**Core ratio in %**	Median (IQR)	40 (20-50)	30 (20-40)	40 (20-50)	30 (20-60)	0.05
**Hospital, n (%)**	UKF	441 (55.6)	0 (0)	99 (83.2)	342 (63.1)	
	UMM	352 (44.4)	132 (100)	20 (16.8)	200 (36.9)	
**DRE, n (%)**	suspicious	214 (27)	33 (25)	32 (26.9)	149 (27.5)	0.9
**Previous biopsies, n (%)**	0	529 (66.7)	83 (62.9)	84 (70.6)	362 (66.8)	0.6
	1	182 (23)	30 (22.7)	27 (22.7)	125 (23.1)	
	2	56 (7.1)	13 (9.8)	5 (4.2)	38 (7.0)	
	≥3	26 (3.3)	6 (4.5)	3 (2.5)	17 (3.1)	
**Comorbidities, n (%)**	Diabetes	36 (4.5)	6 (4.5)	14 (11.8)	16 (3.0)	<0.001
	Immunosuppression	16 (2.0)	0 (0)	10 (8.4)	6 (1.1)	
	Catheter	9 (1.1)	0 (0)	2 (1.7)	7 (1.3)	
	Multimorbidity	33 (4.2)	11 (8.3)	2 (1.7)	20 (3.7)	
	Recurrent UTI	6 (0.8)	0 (0)	3 (2.5)	3 (0.6)	
**Rectal swab prior to biopsy, n (%)**	Yes	32 (4.0)	10 (7.6)	8 (6.7)	14 (2.6)	<0.001
**Urine culture prior to biopsy, n (%)**	Yes	368 (46.4)	132 (100)	31 (26.1)	205 (37.8)	<0.001
**Urine culture positive prior to biopsy, n (%)**	Yes	45 (5.7)	15 (11.4)	3 (2.5)	27 (5.0)	0.9
**Histologically confirmed prostatitis, n (%)**	Yes	266 (33.5)	74 (56.1)	42 (35.3)	150 (27.7)	0.6
**UTI within last 12 months, n (%)**	Yes	9 (1.1)	1 (0.8)	5 (4.2)	3 (0.6)	<0.001
**Antibiotics within last 6 months, n (%)**	Yes	50 (6.3)	16 (12.1)	10 (8.4)	24 (4.4)	<0.001

Descriptive characteristics of 793 patients who underwent transrectal prostate biopsy stratified according to prescribed antibiotic prophylaxis and single dose (intravenous) or multiple doses (oral) application. PSA, initial Prostate Specific Antigen; DRE, Digital rectal examination; UTI, Urinary tract infection; UKF, University Hospital Frankfurt; UMM, University Hospital Mannheim.

### Incidence of Infectious Complications

A single dose of cefotaxime was administered intravenously to 132 patients (16.6%), whereas 119 patients (15%) received multiple oral doses of cefpodoxime. Both groups were compared to 542 patients (68.3%) who received fluoroquinolones either as an intravenous single dose (28.4%) or multiple oral doses (71.6%) prophylaxis. A multiple dose approach was applied for a median of five days (IQR 5-5) for oral cephalosporins and fluoroquinolones.

The total number of patients with severe infectious complications after biopsy was eight (1.0%). One patient per cephalosporine group (0.8% each) and six patients (1.1%) in the fluoroquinolone group reported a complication (p=0.9). The rate of urosepsis was 0.3% (n=2) including one patient in the cefotaxime group and one patient in the fluoroquinolone group. Two patients with an UTI (0.4%) and three patients with a prostatitis (0.6%) received a prophylaxis with fluoroquinolones, one patient with an epididymitis (0.8%) received cefpodoxime. However, there were no significant differences regarding the single infectious complications (p=0.3).

Moreover, according to fever after biopsy, no significant differences were detected between all groups (p=0.6). **Table 2** summarizes infectious complications and treatments.

**Table 2:** Infectious complication related to the antibiotic prophylaxis regimen.

Variable		Overall N=793	Cefotaxime single dose (intravenous) N=132 (16.6%)	Cefpodoxime multiple doses (oral) N=119 (15%)	Fluoroquinolones N=542 (68.3%)	P value
**Duration of antibiotic prophylaxis, days**	Median (IQR)	5 (1-5)	1 (1-1)	5 (5-5)	5 (1-5)	
**Application of prophylaxis, n (%)**	intravenous	176 (22.2)	132 (100)	0 (0)	44 (8.1)	
	oral	577 (72.8)	0 (0)	119 (100)	458 (84.5)	
	Unknown	40 (5.0)	0 (0)	0 (0)	40 (7.4)	
**Infectious complication after biopsy, n (%)**	Yes	8 (1.0)	1 (0.8)	1 (0.8)	6 (1.1)	0.9
**Infectious complication, n (%)**	Epididymitis	1 (0.1)	0 (0)	1 (0.8)	0 (0)	0.3
	UTI	2 (0.3)	0 (0)	0 (0)	2 (0.4)	
	Prostatitis	3 (0.4)	0 (0)	0 (0)	3 (0.6)	
	Urosepsis	2 (0.3)	1 (0.8)	0 (0)	1 (0.2)	
**Fever after biopsy, n (%)**	Yes	5 (0.6)	1 (0.8)	0 (0)	4 (0.7)	0.6
**Treatment of infectious complication, n (%)**	Outpatient treatment	3 (0.4)	0 (0)	1 (0.8)	2 (0.4)	0.3
	Hospital	5 (0.6)	1 (0.8)	0 (0)	4 (0.7)	
**Antibiotic treatment of infectious complication, n (%)**	nonoral	4 (0.5)	1 (0.8)	0 (0)	3 (0.6)	0.4
	oral	4 (0.5)	0 (0)	1 (0.8)	3 (0.6)	
**Duration of infect treatment, days**	Median (IQR)	10 (6-13)	11 (11-11)	5 (5-5)	11 (7-16)	0.7

Antibiotic prophylaxis, infectious complication rates and infect treatment of 793 patients who underwent transrectal prostate biopsy stratified according to the prescribed antibiotic prophylaxis application form. UTI: Urinary tract infection.

## Discussion

Due to the increasing bacterial resistance rates of fluoroquinolones, reported to be up to 50% in Escherichia coli, and the suspended indication for prophylaxis due to rare, but potentially severe side effects (e.g., confusion, arterial aneurysms, tendinopathy), a paradigm shift in antibiotic prophylaxis for prostate biopsies is required (9, 16). We aimed to address this void and revealed several important observations:

First, the results of this large multicenter retrospective analysis of patients undergoing a transrectal prostate biopsy demonstrated a low rate of clinically relevant overall infectious complications (1.0%). Moreover, no significant differences between the usage of cephalosporins vs. fluoroquinolones as a monotherapeutic antibiotic prophylaxis were observed (0.8% *vs.* 1.1%, p=0.9). These observations are noteworthy, since in a recent meta-analysis among 1141 evaluating antibiotic prophylaxis vs. placebo, the rate of infectious complications was 5.6% (10). The lower rate in our cohort might be explained by the definition of infectious complications. We focused exclusively on complications leading to an emergency department visit. Since most studies did not distinguish between the severity of infectious complications, inclusion of e.g., a mild cystitis led to higher rates of overall complications. Importantly, the incidence of complications also depends on geographic regions which results in variation of complication rates from 0-6%, as reported in the systematic review of Roberts et al. (17).

Second, the pathophysiology of post-biopsy infectious complications is explained by two mechanisms: Firstly, flora of the large bowel is directly translocated into the prostate including Escherichia coli as the most frequent causative microorganism (70-90%) and secondly, a bacterial colonization of the prostate or urogenital mucosa before the procedure is considered to cause an UTI afterwards (18, 19). By now, there is an ongoing debate on the optimal alternative non-fluoroquinolone antibiotic regimens to avoid post-biopsy complications. The current European Urology Position Paper on the Prevention of Infectious Complications recommends performing a transperineal biopsy whenever possible (20). If not feasible, three ways of antibiotic prophylaxis for transrectal biopsy are available: i) targeted prophylaxis based on a rectal swab or stool culture, ii) augmented prophylaxis with a combination of at least two different classes of antibiotics or iii) empirical monotherapeutic alternatives to fluoroquinolones (20). Since no superiority of an augmented prophylaxis has yet been demonstrated by ten previous published randomized controlled trials, a monoprophylaxis presents a safe strategy at present. Moreover, since rectal swabs are not available everywhere, an optimal empirical treatment has to be defined. In consequence, our data suggest that third generation cephalosporins, intravenously or orally administered, represent a safe empirical treatment strategy in accordance with the current European Urology Position Paper.

Third, cephalosporins of the third generation, a class of ß-lactam antibiotics, have a broad-spectrum antimicrobial activity against gram-positive, but more relevant gram-negative organisms and are therefore suitable candidates for prostate biopsy prophylaxis. Concerns against their usage have been made since resistancies against ß-lactams in gram-negative pathogens may lead to failure of prophylaxis (21). Nonetheless, the low incidence of severe infectious complications in our cohort strengthens the evidence for the appropriate use of cephalosporins, either orally or intravenously administered, in transrectal prostate biopsy. This observation is in an agreement with four RCTs, which investigated complication rates of cephalosporins (cefuroxime, cefixime or ceftriaxone) vs. fluoroquinolones or piperacillin/tazobactam (22–25). Moreover, in a pooled analysis of three of these studies, including 244 men receiving non-cephalosporins *vs.* 254 men receiving cephalosporins, no statistically significant differences were detected regarding infectious complication and hospitalization rates (RR 0.57, 95% CI 0.12-2.63). Additionally, the same meta-analysis compared 14 studies of non-fluoroquinolones vs fluoroquinolones, where significantly less infectious events were observed with non-fluoroquinolones prophylaxis (8). However, it is of note that consideration of the local resistance patterns increases the safety of cephalosporins since resistance varies widely depending on the geographical region. A rectal swab or stool culture prior to biopsy to detect resistances beforehand and perform a targeted therapy showed significantly lower infection rates compared to an empirical fluoroquinolone prophylaxis (RR 2.1, 95% CI 1.53-2.88) although data on non-fluoroquinolones-targeted prophylaxes is lacking (10). Due to the former institutional standards, only 32 patients had received a rectal swab. None of these patients had an infectious complication, independent of the antibiotic prophylaxis. Those study results and our observations may be indicative for a general performance of rectal swabs.

With respect to other monotherapeutic antibiotic prophylaxis options, the European Commission recommended 2020 a fosfomycin trometamol usage for prophylaxis in men undergoing prostate biopsy (26). Promising results were demonstrated in different meta-analyses, although one large case-control study revealed inferiority compared to ciprofloxacin (27, 28). Consequently, the definite effect of fosfomycin trometamol remains under debate and no recent trial compared cephalosporins vs. fosfomycin trometamol yet. Further trials or network meta-analyses are needed to directly compare the promising results of cephalosporins vs. fosfomycin for transrectal prostate biopsy.

Fourth, we also assessed whether the application type and duration of the cephalosporins affected the occurrence of severe infectious complications. Although urosepsis occurred in one patient in the single dose group and epididymitis in one patient in the multiple doses group, we observed no significant differences between the application types according to overall infections. This result is contradictory to the recent meta-analysis by Pilatz et al. The available studies on fluoroquinolones indicate that a 1-day prophylaxis beginning at least 24 hours prior to biopsy is comparable to a 3-day course, whereas a single-shot prophylaxis less than 24 hours prior to biopsy is inferior compared to a longer course (10). However, this recommendation was not corroborated in a Cochrane review and data mainly relied on fluoroquinolones (29). In consequence, with regard to duration and application type, a comparison to our results cannot be made.

The different antibiotic strategies of both tertiary care hospitals demonstrated comparable efficacy in prevention of severe infectious complications in this study. Whereas from an antibiotic stewardship point of view a single dose prophylaxis is especially beneficial to avoid antibiotic resistances, the advantage of the multiple oral doses’ application might be extended drug levels. Despite pharmacokinetic differences, patients might prefer the oral application over an intravenous access or *vice versa*.

Limitations of this work are firstly the non-randomized retrospective cohort design, which nevertheless increases the knowledge on usage of cephalosporins as a monoprophylaxis due to its large multicenter population size. Second, infectious complications were not assessable from outpatient visits, meaning that mild complications might be underestimated. Moreover, small numbers of infectious complications precluded more complex analyses, such as logistic regression models. Third, definitions of UTI complications were not based on urine cultures but on self-reported symptoms and in-hospital examinations and reports. Finally, despite the large cohort size, it is likely that the low incidence of complication events limited the statistical power of some variables of interest and the majority of patients received fluoroquinolones. Thus, especially the evaluation of other risk factors or comorbidities associated with infectious complications was unfortunately not possible.

## Data Availability Statement

The raw data supporting the conclusions of this article will be made available by the authors, without undue reservation.

## Ethics Statement

The studies involving human participants were reviewed and approved by the Department of Urology, University Hospital Frankfurt (UKF), Germany and the Department of Urology and Urosurgery, University Medical Center Mannheim (UMM).

## Author Contributions

Conceptualization: MW, JH, PM, and NW. Data curation: MW, JH, MNW, SD, BH, CW, PM, and NW. Formal analysis: MW, JH, PM, and NW. Funding acquisition: — Investigation: MW, JH, PM, and NW. Methodology: MW, PM, and NW. Supervision: TH, MK, MM, and FC. Validation: TH, MK, MM, FC, JH, PM, and NW. All authors contributed to the article and approved the submitted version.

## Conflict of Interest

The authors declare that the research was conducted in the absence of any commercial or financial relationships that could be construed as a potential conflict of interest.
